# Supplementary material for: Reconciling Mining with the Conservation of Cave Biodiversity: A Quantitative Baseline to Help Establish Conservation Priorities
Source: PLoS One. 2016 Dec 20;11(12):e0168348. doi: 10.1371/journal.pone.0168348 (PMC5173368; doi:10.1371/journal.pone.0168348)
Supplement: S1 Dataset — (ZIP) [file pone.0168348.s002.zip › Taxa/Serra Norte/SN_2013/N4WS/Lista N4WS-44.pdf]

CAVIDADE N4WS-0044

Classe	Ordem	Fam/Outros	Gên/Outros	Espécie	S-U	U-S	Zon.
Annelida	Oligochaeta			sp.1	4		E
Arachnida	Amblypygi	Phrynidae	*Heterophrynus*	*longicornis*	1		P
Arachnida	Araneae	Oonopidae	aff. *Triaeris*	sp.1		X	E
Arachnida	Araneae	Salticidae		sp.2		X	E
Arachnida	Araneae	Scytodidae	*Scytodes*	*globula*	4		P
Arachnida	Araneae	Theridiosomatidae	*Plato*	sp.1		X	E
Diplopoda	Polyxenida	Hypogexenidae		sp.1		X	E
Insecta	Diptera	Psychodidae	*Sciopemyia*	*sordellii*		X	E
Insecta	Diptera	Tipulidae	Tipulinae	sp.		X	E
Insecta	Hymenoptera	Formicidae	*Pachycondyla*	*striata*		1	E
Insecta	Isoptera	Termitidae	*Nasutitermes*	sp.	X	X	E/P
Insecta	Orthoptera	Phalangopsidae	*Phalangopsis*	sp.	6		P
Amphibia	Anura	Strabomantidae	*Pristimantis*	cf. *fenestratus*	1		E
Mammalia	Chiroptera	Emballonuridae	*Peropteryx*	sp.	3		P
